# Controversies in Thrombolysis

**DOI:** 10.1007/s11910-017-0767-5

**Published:** 2017-06-30

**Authors:** Peter A. G. Sandercock, Stefano Ricci

**Affiliations:** 1 Centre for Clinical Brain Sciences, University of Edinburgh, Chancellor’s Building, Little France Crescent, Edinburgh, EH16 4SB UK; 2 UO Neurologia, USL Umbria 1, Ospedale, Via Angelini 10 - 06012, Citta’ di Castello, PG Italy

**Keywords:** Ischaemic stroke, Thrombolytic therapy, Alteplase, Intracerebral haemorrhage, Functional outcome, Imaging

## Abstract

**Purpose of Review:**

The purpose of the review is to examine recent evidence on the effects of intravenous thrombolysis and identify the remaining uncertainties.

**Recent Findings:**

We review the results of two large trials (the third International Stroke Trial (IST-3) and The Enhanced Control of Hypertension and Thrombolysis Stroke Study (ENCHANTED)) and the publications from the individual patient data analyses of the trials of alteplase conducted by the Stroke Thrombolysis Trialists Collaboration.

**Summary:**

Despite about a 2% risk of fatal intracerebral haemorrhage, on average, adult patients of all ages treated with 0.9 mg/kg alteplase within 4.5 h will have better long-term functional outcome. The use of a lower dose of alteplase (0.6 mg/kg) is associated with a lower risk of haemorrhage but its effect on functional outcome has not been proven to be non-inferior to standard dose therapy. Some clinicians feel confident to treat selected patients who present beyond 4.5 h or have unknown time of onset, but many also agree that the current trials and other research is needed to reliably define the optimum imaging methods and treatment eligibility criteria.

## Introduction

The majority of acute ischaemic strokes are the result of the occlusion of a cerebral artery, though the nature of the arterial obstruction can be very variable. [1] Commonly, large cerebral vessels are occluded by emboli (consisting of thrombus or other material) which reach the cerebral circulation from the chambers of the heart or great vessels while smaller vessels may be occluded by intrinsic vessel wall disease (such as lipohyalinosis, arteritis or other pathologies). As a treatment for acute ischaemic stroke, intravenous administration of fibrinolytic drugs is targeted chiefly on large vessel occlusion and aims to promote lysis of any emboli that contain fibrin. Clot lysis leads to reperfusion of ischaemic brain and should improve the chance of stroke recovery, but this benefit may be negated by an increased risk of intracerebral haemorrhage. We review recent evidence on the factors that influence the balance of benefits and harms of this important treatment.

## Recent Evidence from Large Randomised Controlled Trials

The publication of the results of third International Stroke Trial (IST-3), the largest ever trial of intravenous (iv) thrombolysis with alteplase (0.9 mg/kg), with 3035 patients, doubled the world evidence base on iv alteplase. [2] The study had two unique features; it included a wider range of patients than previous studies, chiefly patients aged over 80 years (*n* = 1617) and followed them for up to 3 years (previous studies followed-up for just 3 months). [3, 4•] These data were then included in the individual patient data meta-analysis of the nine major trials of alteplase conducted by the Stroke Thrombolysis Trialists Collaboration (including 6756 patients**)** [5••]. This review concluded: ‘Irrespective of age or stroke severity, and despite an increased risk of fatal intracranial haemorrhage during the first few days after treatment, alteplase significantly improves the overall odds of a good stroke outcome when delivered within 4·5 h of stroke onset, with earlier treatment associated with bigger proportional benefits’. [5••]

## The Need to Remove the Upper Age Limit for Treatment

The STTC data [5••] prompted the UK drug regulatory agency to suggest that there was a case for the 80-year upper age limit for treatment specified in the EU approval for alteplase to be reconsidered. [6] We strongly support this view, so the product licence, the summary of product characteristics and treatment guidelines should all be aligned to permit treatment of people aged over 80 years.

## What Factors Do and Do Not Influence Risk of Intracerebral Bleeding?

### Age and Time to Treatment

See Table 1. The new data clearly show that neither increasing time to treatment nor increasing age have a significant influence on the ***relative*** risk of intracerebral bleeding [5••, 7••]

**Table 1:** Risk of fatal intracerebral haemorrhage and modelled absolute excess due to alteplase in different subgroups. The absolute excess risk (and its 95% confidence interval (CI)) for each subgroup is estimated by applying the odds ratio among all randomised patients to the average expected risk among control-allocated patients for that subgroup (estimated from a logistic regression model adjusted for trial, treatment allocation, the subgroup of interest and average levels of the other two baseline characteristics) [7••]

	Alteplase (*n* = 3391)	Control (*n* = 3365)	Modelled absolute excess (95% CI)
Treatment delay			
≤3.0 h	22/787 (2.8%)	2/762 (0.3%)	1.6% (0.6–2.6%)
>3 to ≤4.5 h	35/1375 (2.5%)	7/1437 (0.5%)	2.1% (1.1–3.0%)
>4.5 h	34/1229 (2.8%)	4/1166 (0.3%)	1.9% (1.0–2.8%)
Age			
≤80 years	59/2512 (2.3%)	9/2515 (0.4%)	2.0% (1.2–2.8%)
>80 years	32/879 (3.6%)	4/850 (0.5%)	1.7% (0.7–2.7%)
Baseline NIHSS			
0–4	3/345 (0.9%)^1^	0/321 (0.0%)	NE
5–10	20/1281 (1.6%)	5/1252 (0.4%)	1.3% (0.6–1.9%)
11–15	23/794 (2.9%)	1/808 (0.1%)	1.9% (0.9–2.9%)
16–21	24/662 (3.6%)	5/671 (0.7%)	2.6% (1.3–4.0%)
≥22	21/309 (6.8%)	2/313 (0.6%)	4.1% (1.8–6.4%)
All patients	91/3391 (2.7%)	13/3365 (0.4%)	

*NE* not estimable

^1^Note: With an observed frequency of 3 fatal ICH among 345 patients allocated alteplase, the 95% CI for 0.9% proportion with ICH is 0-1.8%

### Stroke Severity

By contrast, baseline stroke severity has a substantial effect; the ***absolute*** risk of fatal intracranial bleeding is lowest in mild stroke and highest in more severe stroke [7••] (Table 1).

### Antiplatelet Therapy

Patients who receive antiplatelet drugs in the 48 h before their stroke onset have a higher risk of intracerebral bleeding, but have the same likelihood of a good functional recovery as those who do not. [8] By contrast, the co-administration of aspirin with the thrombolytic causes a sufficient increase in bleeding to negate any benefit on functional outcome. [9] The Enhanced Control of Hypertension and Thrombolysis Stroke Study (ENCHANTED) trial also suggested that patients already receiving dual antiplatelet therapy might receive greater net benefit with lower dose (0.6 mg/kg) alteplase.

## Absolute Benefit from iv Thrombolysis Depends on Baseline Stroke Severity

**Fig. 1:**
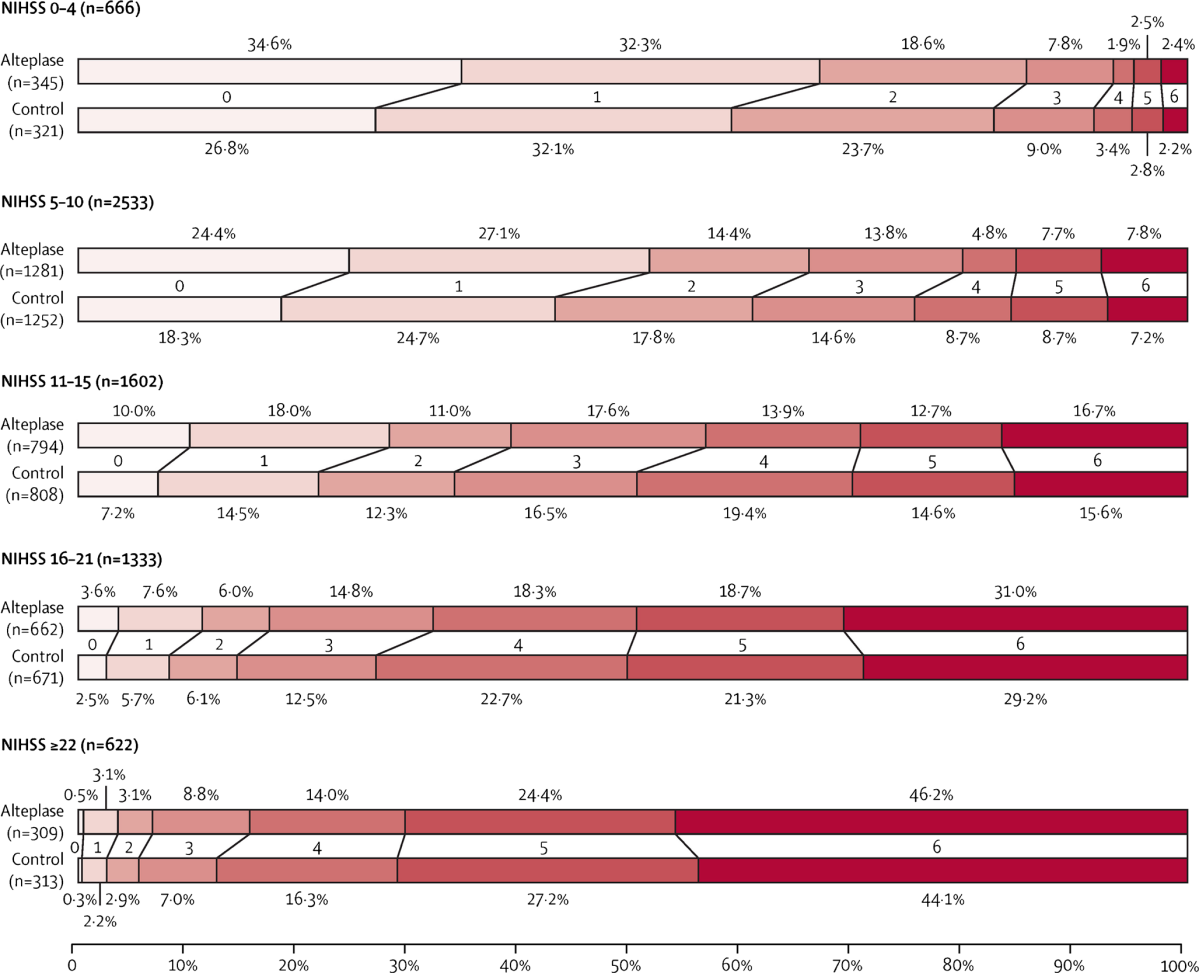
Estimated proportion of patients in each mRS score category with or without alteplase, according to stroke severity at baseline. An mRS of 0–1 indicates an excellent outcome: survival symptom free or with residual symptoms with no loss of activity. mRS 5–6 indicates bed bound or dead at 3–6 months. In IST-3, 125 (4.1%) of 3035 patients died between 3 and 6 months. For comparability of mRS 6 between IST-3 and the other trials (which assessed mRS scores at 3 months), these patients were reassigned an mRS of 5 for this analysis. *NIHSS* National Institutes of Health Stroke Scale, *mRS* modified Rankin Scale, *IST-3* Third International Stroke Trial. Reproduced with permission, Elsevier from Stroke Thrombolysis Trialists Collaboration 2016 [7••]. NNT for mRS 0–1 is 10 within 3 h, 19 from 3 to 4.5 h and 50 from 4.5 to 6 h. Reproduced from Fig. 4 in. Whiteley et al., Risk of intracerebral haemorrhage with alteplase after acute ischaemic stroke: a secondary analysis of an individual patient data meta-analysis. *Lancet Neurol* 2016; 15: 925–33

Figure 1 shows the expected effect of thrombolysis in people with different baseline levels of stroke severity, ranging from mild (National Institutes of Health Stroke Scale (NIHSS ≤5) to very severe (NIHSS >22)). When making the decision about whether to treat or not, it is the absolute risks of fatal bleeding and of a good outcome that matter the most. People with mild stroke will make a good recovery, even without thrombolysis (small absolute benefit), while in severe stroke, the proportion of patients becoming independent in activities of daily living is small and not greatly influenced by treatment (small absolute benefit). However, on average, for many patients with a non-trivial stroke, thrombolysis can be expected to ‘shift’ treated patients of at least moderate severity towards a lesser degree of neurological impairment and disability than those not treated. Even though iv thrombolysis is of net benefit, it is not a cure-all. For example, of patients with a moderate to severe stroke (NIHSS 11–15) treated with iv thrombolysis, one would expect 39% to be alive and independent, but 61% would be expected to die or be dependent on others in activities of daily living. Such patients, who despite thrombolytic therapy are not expected to make a full recovery, may then be candidates for endovascular therapy (e.g. mechanical clot retrieval with a stent retriever device). [10] In our view, the priority is to ensure all patients with non-trivial strokes are considered for iv thrombolysis and all eligible patients treated as promptly as possible. There is debate as to whether or not it is necessary to give iv thrombolysis before endovascular treatment (we think it is), but ongoing trials such as SWIFT DIRECT will help to answer the question.

## Controversies About Patient Selection

### Mild Stroke

Patients with baseline NIHSS can be expected to do well without treatment; however, if the stroke causes a deficit such as isolated hemianopia or isolated aphasia, the NIHSS will be low, and the person’s quality of life may be very adversely affected. Yet, treatment plausibly carries a 1% risk of fatal intracranial bleeding, and hence the discussions with the patient and the family to weigh up potential benefits and harms may be very difficult because of the uncertainties. As a result, two trials comparing thrombolysis with control in patients with mild stroke are ongoing to resolve the uncertainty, PRISMS and TEMPO-2 (Table 2).

**Table 2:** Major ongoing trials of intravenous thrombolysis versus control

Trial type and name	Trial acronym	Target population	Intervention	Sample size	Study registration details
Mild stroke					
A Study of the Efficacy and Safety of Alteplase in Participants with Mild Stroke	PRISMS	Mild stroke, NIHSS <5, <3 h	iv alteplase	313	https://clinicaltrials.gov/ct2/show/record/NCT02072226
A Randomised Controlled Trial of TNK-tPA Versus Standard of Care for Minor Ischemic Stroke With Proven Occlusion	TEMPO-2	Mild stroke with large vessel occlusion	iv tenecteplase	1274	https://clinicaltrials.gov/ct2/show/NCT02398656
Wake-up stroke/unkown time of onset/late entry					
Tenecteplase in Wake-up Ischaemic Stroke Trial	TWIST	Wake up stroke selected by CT, CTA	iv tenecteplase	500	http://www.isrctn.com/ISRCTN10601890
Wake-up	WAKE-UP	MRI DWI-FLAIR-mismatch	iv alteplase	800	https://clinicaltrials.gov/ct2/show/NCT01525290
Japanese THrombolysis Acute Wake-up and Unclear-onset Stroke Trial	THAWS	MRI DWI-FLAIR-mismatch	iv alteplase	300	https://clinicaltrials.gov/ct2/show/NCT02002325
Fourth European Cooperative Acute Stroke Study	ECASS-4	Penumbral mismatch imaging + perfusion volume (PWI) to infarct core (DWI) ratio of ≥1.2, and a minimum perfusion lesion volume of 20 ml	iv alteplase	264	http://www.isrctn.com/ISRCTN71616222
Safety and Efficacy of Alteplase When Administered in Chinese Patients with Acute Ischemic	N/A	CT or MR exclusion of intracranial haemorrhage	iv alteplase	120	https://clinicaltrials.gov/ct2/show/NCT02930837
Hemispheric Stroke Where Thrombolysis is Initiated Between 3 and 4.5 Hours After Stroke Onset					
Older patients					
Treatment Elderly Stroke Patients in Italy (aged >80) within 3 h	TESPI	CT or MR exclusion of intracranial haemorrhage	iv alteplase	300	N/A

This table only includes trials of iv alteplase versus control and excludes trials of alteplase versus other comparators**,** combinations with antithrombotic agents or endovascular procedures

### Severe Stroke

Some individuals with severe stroke may be prepared to accept a high (4%, or 1 in 25) immediate risk of fatal haemorrhage from treatment if their prognosis is poor (the person has ‘nothing to lose’), whereas for others, that level of risk may be unacceptably high. Better clinical decision aids are needed to support clinicians and patients in dealing with the difficult discussions that can arise in these circumstances. [7••]

### Choice of Imaging Modality for Routine Cases

Facilities and local practice in the use of imaging to select patients for reperfusion therapy varies widely around the world. [11] For routine cases of stroke, where the time of onset is known reliably and treatment can be given within 4.5 h, we have a strong personal preference for rapid non-contrast CT as the imaging method of first choice to exclude haemorrhage, and if the stroke is moderate to severe, and endovascular therapy is rapidly available locally, CT angiography (CTA) is in addition. Demchuk et al. argue that CTA should become the minimum standard of care in acute disabling ischaemic stroke. [12]

### Imaging for Endovascular Therapy, Late-Presenting (>4.5 h), Wake-up and Unknown Time of Onset Stroke

This is a controversial topic; criteria vary enormously and only 1% meet a common set of criteria. [13] In well-resourced centres where multimodal MR scanning is available, some experts feel confident to select cases for treatment on the basis of advanced imaging findings. However, there is no consensus on precisely what the imaging selection parameters for treatment should be and there is no ‘clear winner’. Current trials aim to better define the criteria for patient selection and given the limited availability of multimodal MR in some sites, the TWIST trial is evaluating the use of CT and CT angiography in wake-up stroke (Table 2). The DEFUSE 3 trial of endovascular therapy versus control also permits the use of either CT or MR perfusion and angiography for case selection. [14] When CTA and MRA are not available, transcranial Doppler ultrasound, in experienced hands gives a good indication of vessel patency [15].

### Patients on Oral Anticoagulants

Depending on age, one sixth to one third of all acute ischaemic stroke patients are in atrial fibrillation, and many are on long-term oral anticoagulants when they arrive at the hospital, which creates problems for clinicians considering thrombolytic or endovascular therapy.[16, 17] Rapid reversal of Vitamin-K based anticoagulation to achieve an INR of <1.7 to permit iv thrombolysis is difficult [16]. The situation for patients treated with the novel direct oral anticoagulants (DOACs) is changing rapidly with the introduction into clinical practice of agents that can rapidly reverse the direct thrombin inhibitor dabigatran (idarucizumab). A small case series suggests that reversal of dabigatran prior to iv thrombolysis is safe [18], and Diener and others have proposed a management algorithm to handle such cases. [17] The agent that reversed factor X (adexanet) will be available in the near future.

### Dose of Alteplase

Is a lower dose of iv alteplase preferable? The ENCHANTED study compared standard dose (0.9 mg/kg) with lower dose (0.6 mg/kg) alteplase [19]. Major symptomatic intracerebral haemorrhage occurred in 1.0% of the participants in the low-dose group and in 2.1% of the participants in the standard-dose group (*P* = 0.01). However, in terms of the effect on the proportion dead or disabled at 90 days, the lower dose did not meet the criteria for noninferiority. [19] The lower dose is widely used in Asia, and as a result of the trial, many clinicians will continue to use the lower dose because of fears of haemorrhage, despite the potential loss of efficacy. By contrast, in the West, clinicians may well tend to continue with the guideline approved standard dose, unless there is a particular concern about bleeding risk. [20]

### Planning Service Delivery

There has been much debate about how best to organise stroke services to deliver reperfusion therapy to ensure the largest number of patients benefit. Most strokes occur in low and middle income countries, where delivery of basic acute stroke care is challenging [21] and beyond the scope of this article. As a result, the use of iv thrombolysis varies enormously around the world. In a systematic review of worldwide use of thrombolysis for stroke Berkowitz found that, of 214 countries and independent territories, 64 (30%) reported use of intravenous the average percentage treated tissue plasminogen activator for acute ischemic stroke; in the medical literature is 3% (1/36) of low-income, 19% (10/54) lower-middle-income, 33% (18/54) of upper-middle-income and 50% (35/70) of high-income-countries (test for trend, *P* < 0·001). [22] Within high-income countries like the USA, the proportion treated varies geographically [23], and treatment is not delivered to all that might benefit, though treatment rates are improving [24]. To deliver IVT/EVT equitably and affordably across a region or a country must be adapted to local resources will be a challenge, even for advanced economies, [23] and is beyond the scope of this article.

## Conclusions

These new data confirm clearly that ‘time is brain’ and that the priority remains rapid recognition of stroke, transport to hospital with a fast-track stroke thrombolysis system of clinical and radiological assessment followed by prompt treatment with iv thrombolysis where appropriate or inclusion in a relevant trial designed to resolve one of the current uncertainties. These goals are best achieved within a well organised and dedicated stroke unit.
